# A Single Gene Coordinates the Social Life of Honeybees

**DOI:** 10.1371/journal.pbio.0050086

**Published:** 2007-03-06

**Authors:** Liza Gross

Students of the evolution of social behavior got a big boost with the publication of the newly sequenced honeybee genome in October 2006. The honeybee*(Apis mellifera)*belongs to the rarified cadre of insects that pool resources, divide tasks, and communicate with each other in highly structured colonies. Understanding how this advanced state of organization evolved from a solitary lifestyle has been an enduring question in biology.

In a new study, Mindy Nelson, Kate Ihle, Gro Amdam, and colleagues reveal one possible path to community by showing that a single gene controls multiple traits related to honeybee sociability. First characterized for its role in reproduction, the gene,*vitellogenin*, is widely found in egg-laying insects, which depend on it for egg cell development.

A honeybee’s lot in life depends on its age, gender, and caste. Reproduction falls to the queen and drones, while essentially infertile females, the workers, perform all the other duties required to support the colony. As young adults, workers tend larvae and perform assorted tasks in the hive. After about three weeks, they switch from domestic chores to foraging, and eventually specialize in pollen or nectar collection.

Scientists began to suspect that the protein synthesized from the*vitellogenin*gene—vitellogenin—might affect these social life history traits in honeybees as it became clear that the protein supported an array of functions not directly linked to egg-laying. For example, sterile workers synthesize vitellogenin to make the royal jelly they feed larvae. It can also prolong the lifespan of both workers and the queen by reducing oxidative stress.

As bees undergo the complex behavioral shift demanded by the change in job description, their physiology changes too: they have higher levels of juvenile hormone and lower levels of vitellogenin. It was speculated that these two physiological factors repress each other to affect the bees’ behavior, with vitellogenin repressing juvenile hormone in younger bees to inhibit the shift from nest to field, and juvenile hormone repressing vitellogenin in bees that have switched to foraging to ensure that they stay true to their task and do not revert to nest jobs. In a previous study, the researchers also proposed that changes in*vitellogenin*gene expression early in life could foster the selective behavior that creates the division of labor between pollen and nectar specialists.

To test these proposed roles of vitellogenin in coordinating the social life of the honeybee, Nelson et al. inhibited the expression of the*vitellogenin*gene with RNA interference (RNAi). This gene-silencing tool introduces a double-stranded RNA (dsRNA) product whose sequence is complementary to a target gene, thereby setting off a series of events that ultimately “knocks down” the target gene. The researchers injected a*vitellogenin*dsRNA preparation into the abdomen of a subset of bees and compared their behavior and lifespan to a control group. (The control group also received a dsRNA treatment designed to mimic the stress of experimental handling without affecting gene expression.) The bees’ vitellogenin levels were monitored at 10 days, 15 days, and 20 days old to make sure the RNAi effects persisted.

**Figure:**
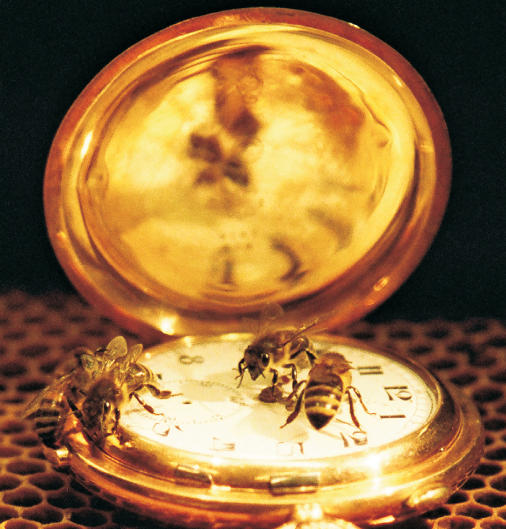
A honeybee gene originally used in egg production has become an important behavioral modulator and a timekeeper of social life. (Image: Siri-Christine Seehuus)

Compared to controls, dsRNA-treated bees had consistently lower levels of vitellogenin protein. These*vitellogenin*“knockdowns” started foraging at a younger age than controls—confirming that vitellogenin affects workers’ occupational fate by repressing the shift from domestic to foraging tasks. The foragers also showed a preference for nectar, in keeping with evidence that workers genetically predisposed toward nectar have lower vitellogenin levels before leaving the nest, while those predisposed toward pollen have higher levels. But more directly, the researchers argue, these results show that vitellogenin controls social foraging specialization. What’s more, the vitellogenin-deficient bees died earlier than the controls, demonstrating the protein’s influence on honeybee longevity.

Altogether, these results demonstrate that vitellogenin regulates the organizational structure of honeybee society by influencing workers’ division of labor and foraging preference. Vitellogenin, the researchers conclude, controls not only when bees start foraging and how long they live, but what they forage. Higher levels early in life favor pollen; lower levels favor nectar. Since current methods cannot yet distinguish the effects of vitellogenin from those of juvenile hormone, the researchers argue that the two physiological factors should be considered as partners in mediating task assignment and specialization. Since this partnership is uncommon in insects, it suggests that social behavior in honeybees emerged from a makeover of relations between vitellogenin and juvenile hormone. It also bolsters the notion that factors normally in control of female reproduction can lay the foundation for the transition from solitary life to complex social behavior.

